# A model of speech production based on the acoustic relativity of the vocal tract

**DOI:** 10.1121/1.5127756

**Published:** 2019-10-17

**Authors:** Brad H. Story, Kate Bunton

**Affiliations:** Speech, Language, and Hearing Sciences, University of Arizona, Tucson, Arizona 85721, USA

## Abstract

A model is described in which the effects of articulatory movements to produce speech are generated by specifying relative acoustic events along a time axis. These events consist of directional changes of the vocal tract resonance frequencies that, when associated with a temporal event function, are transformed via acoustic sensitivity functions, into time-varying modulations of the vocal tract shape. Because the time course of the events may be considerably overlapped in time, coarticulatory effects are automatically generated. Production of sentence-level speech with the model is demonstrated with audio samples and vocal tract animations.

## I. INTRODUCTION

Speech production is often viewed as a process of planning and executing articulatory movements that generate an acoustic signal comprised of a temporally ordered stream of phonetic segments. Movement of the articulators is coordinated, or coarticulated, so that multiple segments overlap in time, thus facilitating rapid and efficient transmission of a message (cf. Kent and Minifie, 1977). Models of speech production are typically designed to emulate this process where the movements of the tongue, jaw, lips, velum, and larynx, or some lower dimensional representation of articulation, are orchestrated to collectively form the time-varying shape of the vocal tract, and transform the voice source into speech (cf., Mermelstein, 1973; Coker, 1976; Rubin *et al.*, 1981; Maeda, 1990; Browman and Goldstein, 1992; Story, 2005, 2009, 2013; Toutios *et al.*, 2011).

In contrast, Story and Bunton (2017) proposed a method, in part inspired by the distinctive region model of Mrayati *et al.* (1988), in which an utterance is planned by specifying directional changes of the resonance frequencies relative to those of the underlying vocal tract configuration. When associated with a temporal “event” function, the specified resonance deflections are transformed, via calculations of acoustic sensitivity functions, into a time-varying modulation of the vocal tract. An advantage of this approach is that an explicit specification of vocal tract characteristics such as constriction location is not required. Rather, the model itself finds a time-dependent vocal tract deformation pattern, containing constrictions and synergistic expansions, that results in the specified acoustic goal.

A limitation of the study reported by Story and Bunton (2017) was that the resonance deflection approach was applied only to stop consonants, whereas the modulation of the vocal tract to produce the underlying vowel substrate was independently generated by a kinematic model (Story, 2005). The aim of the present study was to demonstrate that the resonance deflection modeling approach can be used to generate sentence-level speech where all consonants and vowels are specified as a temporal sequence of *relative* acoustic events, partially overlapped in time, and then transformed to time-varying vocal tract modulations that automatically contain the effects of coarticulation. The scope of the study is limited to description of the vocal tract model and demonstration of sentence-level synthesis. Comparison of the model output to articulatory data and formal perceptual evaluation of the synthesis will be the focus of future research.

## II. VOCAL TRACT MODEL CONTROLLED BY RELATIVE ACOUSTIC EVENTS

The structure of the model used in this study was essentially the same as described in Story and Bunton (2017). A time-varying vocal tract area function is generated as the product of a neutral configuration Ω(*i*) and a deformation function *D*(*i*,*n*),

$$A(i,n)=\Omega (i)D(i,n),\;i=[1,N_{x}],\;n=[1,N_{d}],$$ (1)

where, at any given time instant *n*, *A*(*i*,*n*) consists of *N_x_* = 44 contiguous sections or tubelets, each with a length of *L*(*i*) = 0.396 825 cm. Although it is not suggested that this level of accuracy is required for the section length, the number is dictated by the wave propagation algorithm used in this study to synthesize speech (Liljencrants, 1985; Story, 1995) such that *L*(*i*) is equal to the speed of sound (*c *=* *35 000 cm/s) divided by two times the sampling frequency (*F_s_* = 44 100 Hz). The actual distance from the glottis corresponding to the *i*th section is then $x(i)=\sum_{z=1}^{i}L(z)$, and results in an overall tract length of 17.46 cm (this length is simply an example used for this study; it could be set to any value appropriate for a human vocal tract by using a different number of tubelet sections or alternate sampling frequency). The time dimension is represented by *n*, and the total duration of a given utterance is *N_d_* samples.

The shape of the deformation function *D*(*i*,*n*) in Eq. (1) is controlled by three parameters representing the polarity and normalized magnitude of the resonance deflections required to generate a specific phonetic target. These control parameters are denoted *δ*_1_, *δ*_2_, and *δ*_3_, and can be assigned any value between −1 and 1. When written in a vertical orientation, they form a resonance deflection pattern (RDP) that coincides with the spatial arrangement of formants as observed in a spectrogram. For example, in the equation below,

$$\left[\begin{array}{c} \delta_{3}\\ \delta_{2}\\ \delta_{1} \end{array}\right]=\overset{∼\text{bilabial}}{\left[\begin{array}{c} -1\\ -1\\ -1 \end{array}\right]}\text{or}=\overset{∼\text{alveolar}}{\left[\begin{array}{c} 1\\ 1\\ -1 \end{array}\right]}\text{or}=\overset{∼\text{velar}}{\left[\begin{array}{c} -1\\ 1\\ -1 \end{array}\right]},\;$$ (2)

the first RDP vector would indicate a downward deflection of all three resonances, typical of a bilabial consonant, whereas the other two RDPs are representative of alveolar and velar consonants, respectively (cf. Story and Bunton, 2017).

Each RDP must be associated with an *event* function *E*(*n*) that dictates the time course of the resulting vocal tract modulation required to actually produce the acoustic/phonetic event as a speech signal. This is a smoothly varying curve whose amplitude is constrained to be between 0 and 1. The event functions used for this study were based on a Gaussian pulse shape,

$$E(n)=e^{-\text{ln}\;(16){\left(\left(n-N_{p}\right)/N_{w}\right)}^{2}},\;n=[1,N_{d}],$$ (3)

where *n* is the current time sample, *N_p_* is the time sample at which a peak amplitude of 1.0 is achieved, and *N_w_* is the width of the Gaussian at half maximum (i.e., at an amplitude of 0.5). The total duration of the event is *N_d_* time samples, the same as in Eq. (1). The sampling frequency of the vocal tract modulation was set to *f_svt_* = 146 Hz, which is the same as the x-ray microbeam database (Westbury, 1994), and will facilitate efficient comparison of the model output to articulatory data in future studies. Thus, the actual time values represented by the parameters in Eq. (3) are $t_{p}=N_{p}/f_{\mathrm{svt}},t_{w}=N_{w}/f_{\mathrm{svt}}$ and $t_{d}=N_{d}/f_{\mathrm{svt}}$.

## III. TRANSFORMATION OF RESONANCE DEFLECTION PATTERNS INTO VOCAL TRACT MODULATION

Processing steps for transforming the RDPs (i.e., *δ_j_*) associated with an event function *E*(*n*) into a time-dependent vocal tract deformation function *D*(*i*, *n*) will be described in Secs. III A–III D below, first for the case of a *single* specified acoustic event, and then for multiple events, as are required to produce sentence-level speech.

### A. Sensitivity function calculation

**FIG. 1.**
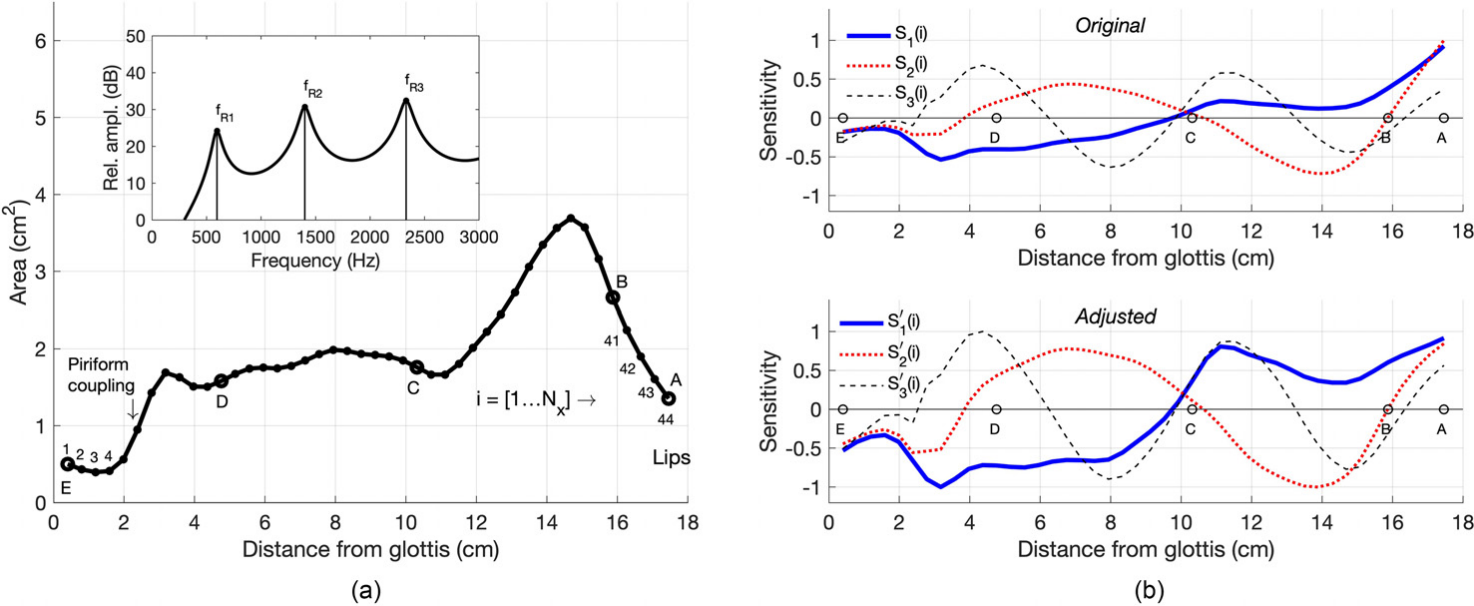
(Color online) Area function and sensitivity functions for the neutral vocal tract Ω(*i*). The points marked with A–E are the approximate locations of the lips (A), incisors (B), hard-palate/soft-palate junction (C), superior aspect of the epiglottis (D), and glottis (E), respectively. (a) Area function for Ω(*i*) is shown with a solid line and dots; each dot represents the *i*th cross-sectional area; inset plot shows the frequency response where peaks are the vocal tract resonances. (b) Sensitivity functions calculated with Eqs. (4) and (5) that correspond to the first three resonances of the area function.

The first step is to calculate the frequency response of the neutral area function Ω(*i*) [see Eq. (1)], and from it determine the resonance frequencies *f_R_*_1_, *f_R_*_2_, and *f_R_*_3_. The specific Ω(*i*) used for this study is shown in Fig. 1(a) and is based on the adult male model described in Story (2005) and Story *et al.* (2018). The piriform sinuses are represented as a single side branch and are coupled to the main vocal tract at 2.4 cm from the glottis (Story, 1995; Dang and Honda, 1997). The frequency response is shown in the upper inset plot where the first three resonances are located at 596, 1401, and 2331 Hz. The sensitivity of each resonance frequency, *f_Rn_*, to a change in vocal tract cross-sectional area is the difference of kinetic energy (*K_e_*) and potential energy (*P_e_*) within each *i*th section, divided by the total energy in the system (e.g., Fant and Pauli, 1975). A sensitivity function can be written as

$$S_{j}(i)=\frac{K_{e_{j}}(i)-P_{e_{j}}(i)}{\sum_{i=1}^{N_{x}}\left[K_{e_{j}}(i)+P_{e_{j}}(i)\right]},\;j=1,2,3;\;i=[1,N_{x}],$$ (4)

where *j* is the resonance number. The kinetic and potential energies, *K_e_* and *P_e_*, for each resonance frequency are based on the pressure $p_{j}(i)$ and volume velocity $u_{j}(i)$ computed for each section of an area vector. These quantities, along with the frequency response function [Fig. 1(a)], were calculated with a transmission-line type model of the vocal tract (Sondhi and Schroeter, 1987; Story and Bunton, 2017) that included energy losses due to yielding walls, viscosity, heat conduction, and acoustic radiation at the lips. The sensitivity functions calculated for Ω(i) are shown in the upper panel of Fig. 1(b) where the solid, dotted, and dashed lines indicate the sensitivity of the first, second, and third resonance frequencies ($f_{R1},\;f_{R2},\;f_{R3}$), respectively, to a small perturbation of the area function, ΔΩ(i). This relation can be written as

$$\frac{\Delta f_{\mathrm{Rj}}}{f_{\mathrm{Rj}}}=\sum_{i=1}^{N_{x}}S_{j}(i)\frac{\Delta \Omega (i)}{\Omega (i)},$$ (5)

where *j* is again the resonance number. Equation (5) dictates that an upward shift in the resonance frequency will occur when a positive change in area, ΔΩ(i)>0, is imposed at values of *i* where $S_{j}(i)>0$, or when a negative change in area, ΔΩ(i)<0, is imposed where $S_{j}(i)<0$; the opposite shift in resonance frequency occurs if the polarities of ΔΩ(i) and $S_{j}(i)$ oppose each other.

### B. Adjustments to the sensitivity functions

To avoid disproportionate influence of any particular region of the vocal tract on the deformation function, the second step is an adjustment that balances the magnitude of each sensitivity function *S_j_*(*i*) from the glottis to the lips. The adjustment is carried out by first storing the polarities of each *i*th section of the *j*th sensitivity function in a vector such that *Q*(*i*) = 1 for $S_{j}(i)\geq 0$ and Q(i)=−1 for $S_{j}(i)<0$, where $i=[1,N_{x}]$. Next, $\left|S_{j}(i)\right|$ is low-pass filtered (second order Butterworth) with a normalized cutoff frequency of 0.1, assigned to a vector *R*(*i*), and then used to generate the trend function $R_{o}(i)=R(i)+\text{max}[|S_{j}(i)|-R(i)]$. An intermediate adjusted sensitivity function is determined by removing the trend such that $R_{a}(i)=(|S_{j}(i)|/R_{o}(i))Q(i)$, where the multiplication by *Q*(*i*) restores the polarity of each section to be the same as the original *S_j_*(*i*). The final adjusted and normalized sensitivity function is $Z_{j}(i)=R_{a}(i)/\text{max}(|R_{a}(i)|)$. Note that the $Q(i),\;R(i),\;R_{o}(i),$ and *R_a_*(*i*) are not assigned *j* indices because they are all temporary vectors used only during the adjustment process of each *j*th sensitivity function.

### C. Calculation of the deformation function

A linear combination of the three sensitivity functions from the previous step can now be formed as

$$y(i)=\delta_{1}Z_{1}(i)+\delta_{2}Z_{2}(i)+\delta_{3}Z_{3}(i),$$ (6)

where the coefficient weights are the *δ_j_* components of the specified RDP vector, and determine the relative contribution of each sensitivity function to the overall shape of *y*(*i*). The deformation function, *D*(*i*, *n*), at each time sample *n* can now be formed by normalizing *y*(*i*) relative to its minimum value, and multiplying by *E*(*n*),

$$D(i,n)=\frac{-\mu E(n)y(i)}{\underset{i\in [1,N_{x}]}{\text{min}}y(i)},$$ (7)

where the minus sign is needed to negate the effect of the denominator always being less than zero. The *μ* parameter controls the *degree* to which the deformation constricts the vocal tract; if *μ* < 1, constrictions will only partially occlude the tract as is characteristic of vowels, liquids, glides, and fricatives; when *μ* = 1 a complete closure will be formed at the location of the minimum value in *y*(*i*); and if *μ* > 1 the extent of the complete closure will spread along the length axis of the vocal tract. The final operation is to use Eq. (1) to generate the composite time-varying area function *A*(*i*,*n*) from the product of Ω(i,n) and *D*(*i*,*n*).

### D. Sequencing multiple acoustic events

Word and sentence-level speech can be generated by sequencing multiple acoustic events along a time axis. Because there may be considerable temporal overlap of the event functions, some additional considerations are needed to generate a deformation function. At every time sample *n*, the steps described previously in Secs. III A–III C are executed in a loop where each iteration *k* attends to one event function. The order of execution is carried out in ascending order of the values of *μ*, and the output of each iteration replaces the original Ω(*i*) vector. That is, when multiple event functions are specified, the RDP associated with the smallest *μ* is used to generate the initial deformation at a given time sample producing a “temporary” $A_{k}(i,n)$ which is then fed back through each of the three steps in Secs. III A–III C, where the next iteration will attend to the RDP with next smallest *μ* value.

## IV. Sentence-level speech production

In this section, use of the vocal tract model to produce sentence-level speech is demonstrated by generating synthetic versions of “*a dog ate a bug*” and “*a frog ate a fly*.” The first sentence contains only vowels and stop consonants, whereas the second includes the added complexity of fricative-liquid clusters.

### A. Sentence 1

**FIG. 2.**
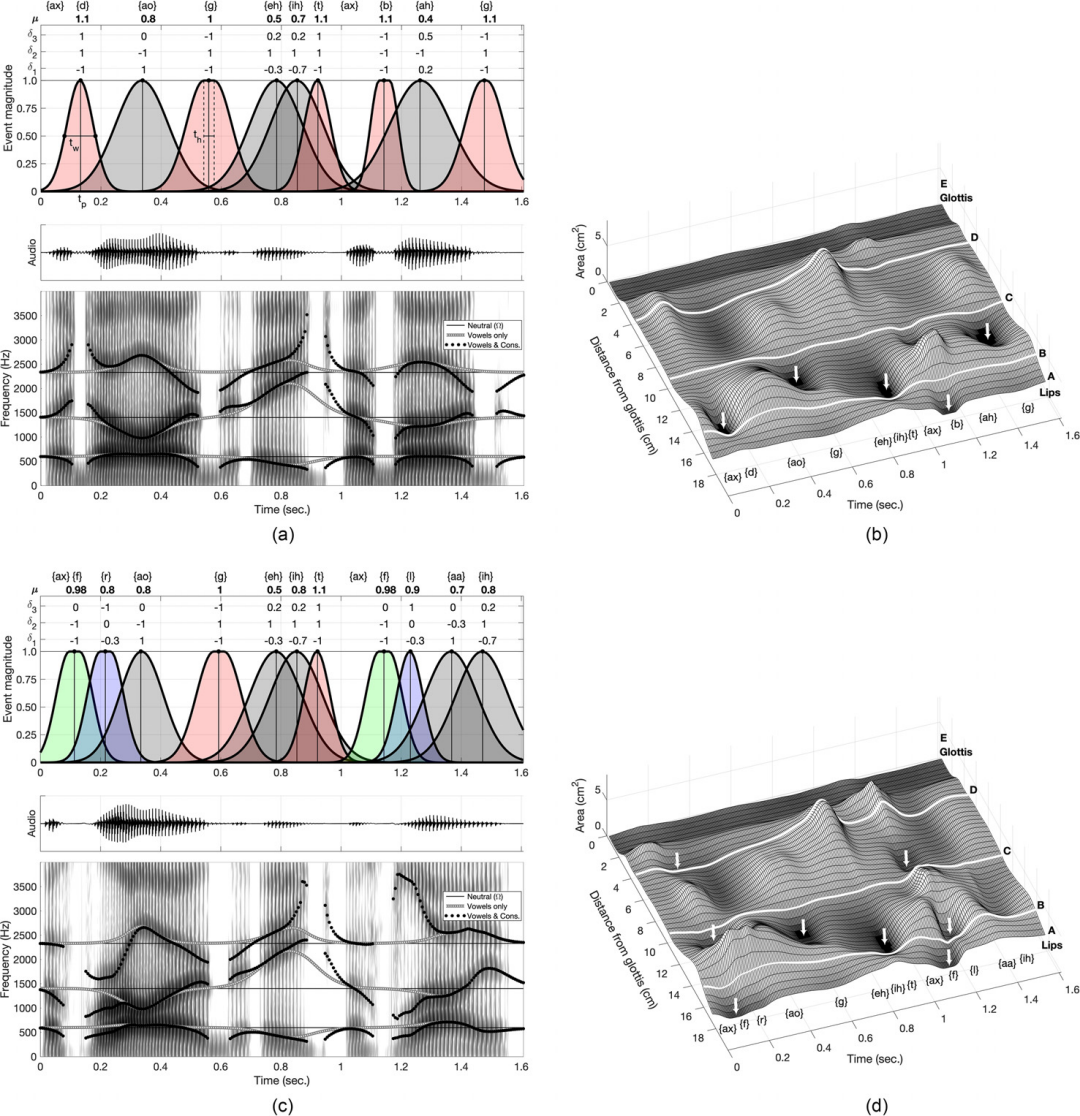
(Color online) Specification of events to produce two sentences and model output. (a) RDPs, event functions, waveform, spectrogram, and calculated vocal tract resonances for the sentence “*a dog ate a bug*.” Overlap of the events is indicated by darkened shading. The *t_p_*, *t_w_*, and *t_h_* are examples of temporal values of peak, width, and hold, respectively. (b) 3 D surface plot of time-varying area function *A*(*i*, *n*) where the arrows mark the stop consonant constrictions and the points marked with (A)–(E) are the anatomic landmarks described in the text and caption for Figs. 1(c) and 1(d) are identical to (a) and (b) but for the sentence “*a frog ate a fly*.”

The RDPs, *μ* values, and event functions for “*a dog ate a bug*” are shown positioned sequentially along a timeline in the upper panel of Fig. 2(a). Distributed along the top of the plot are the ARPAbet phonetic symbols (Shoup, 1980; Klatt, 1987) associated with each acoustic event (the unconventional curly brackets are used here to differentiate vocal tract area functions and calculated resonance frequencies produced by a model, from actual prescribed phonetic targets or transcriptions of real or synthetic talkers). An exception is the unstressed neutral vowel {ax} which is expressed in the output signal simply by the absence of any other specified event. An {ax} occurs at the beginning of the sentence, and again at about 1.05 s. The peak of the first event, corresponding to {d}, occurs at *t_p_* = 0.14 s, and has a half-width *t_w_* = 0.1 s [see Eq. (3)]. The RDP associated with the first event specifies a downward deflection of the first vocal tract resonance and upward deflections of the second and third resonances, respectively. The value of *μ* is 1.1 which assures that the vocal tract will be fully occluded when the event function reaches its peak, and the occlusion will spread spatially along the vocal tract axis because *μ* > 1.

The second event in the sequence, whose peak is located at 0.34 s, is intended to be the vowel {ao} (“aw”) and has an RDP that directs the first resonance upward in frequency and the second resonance downward. The deflection of the third resonance is left unspecified as indicated by *δ*_3_ = 0; this does not mean that *δ*_3_ must always be zero for this vowel, but was deemed sufficient for this particular case. With its location in time and a width of *t_w_* = 0.21 s, the {ao} event function generates considerable temporal overlap with the previous {d} event, as well as with the subsequent {g} event, as can be seen by the darker shading in the figure. During these intervals of overlap, the multiple-event sequencing process described in Sec. III is used to determine the vocal tract area configuration at each point in time.

The next seven events are specified in similar fashion where the *μ* values for the stop consonants are 1.0 or greater and the vowels are less than 1.0. It can be noted that the event function for the {g} includes a period of time, denoted as *t_h_*, where the peak value is held constant at 1.0 in order to sustain the occlusion; this is not a necessary condition to produce {g} but was useful in the timing of the events for this sentence. The extensive temporal overlap of the {eh} and {ih} vowels (peaks located 0.79 and 0.86 s, respectively) produces the diphthong in the word “ate,” which is, in turn, heavily overlapped with the event function for the {t}. The final three events generate “bug,” again with extensive overlap in time. The timing parameters for all specified events are given in Table I.

**TABLE I.** Timing parameters *t_p_*, *t_w_*, and *t_h_* for the two sentences. All values are in seconds but can be converted to samples by multiplying by *f_svt_* = 146.

Sentence 1	“*a dog ate a bug*”										
Event number	1	2	3	4	5	6	7	8	9		
Symbol	d	ao	g	eh	ih	t	b	ah	g		
*t_p_* (s)	0.14	0.34	0.55	0.79	0.86	0.92	1.13	1.27	1.47		
*t_w_* (s)	0.10	0.21	0.14	0.21	0.21	0.10	0.07	0.24	0.14		
*t_h_* (s)	0	0	0.03	0	0	0	0.03	0	0.01		
Sentence 2	“*a frog ate a fly*”										
Event number	1	2	3	4	5	6	7	8	9	10	11
Symbol	f	r	ao	g	eh	ih	t	f	l	aa	ih
*t_p_* (s)	0.10	0.21	0.34	0.58	0.79	0.86	0.92	1.13	1.23	1.37	1.47
*t_w_* (s)	0.10	0.10	0.17	0.14	0.21	0.21	0.10	0.10	0.10	0.21	0.21
*t_h_* (s)	0.03	0.03	0	0.03	0	0	0	0.03	0	0	0

Collectively, the relative acoustic events specified for the sentence generate the time-varying vocal tract area function *A*(*i*, *n*) shown in Fig. 2(b). The lips are labeled as point A, the glottis as point E, and the white lines labeled B, C, and D indicate the approximate anatomic landmarks of the incisors, hard-palate/soft-palate junction, and superior aspect of the epiglottis, respectively. At every point in time, the shape of the area function is influenced by multiple events, and thus represents the coarticulation of the phonetic segments. The complete occlusions indicated by the arrows in the figure are located at points along the vocal tract length that are fairly typical for the bilabial, alveolar, and velar stop consonants they are intended to produce, even though their specification was based entirely on relative deflections of the vocal tract resonances.

Using an algorithm to calculate wave propagation in the vocal tract coupled with a kinematic model of vocal fold vibration (cf. Story, 2013), the *A*(*i*,*n*) in Fig. 2(b) produced the speech signal plotted in the middle panel of Fig. 2(a). The input parameters of the vocal fold model were set to generate a rising and falling fundamental frequency contour, and an abductory maneuver to assure that the {t} in “ate” was unvoiced in the output signal. Aspiration noise produced by glottal turbulence was emulated by adding a noise component to the glottal flow when the Reynolds number within the glottis exceeded a threshold value (Story, 2013). The noise component of the flow was generated in the form proposed by Fant (1960) such that

Unois={Nf(Re2−Rec2)(1×10−6)for  Re>Rec0for  Re≤Rec (8)

where *N_f_* is a broadband noise signal (random noise generated with values ranging in amplitude from −0.5 to 0.5) that has been band-pass filtered between 500–2500 Hz (second order Butterworth), *Re* is the calculated Reynolds number, and *Re_c_* = 1200 is the threshold value below which no noise is allowed to be generated. A similar noise source is used in the vocal tract where the Reynolds number is calculated in each *i*th section at every time sample *n*, and if it exceeds the threshold value *Re_c_*, noise is switched on at a location immediately downstream of that point (cf. Flanagan, 1972, p. 54).

The corresponding wideband spectrogram is shown in the bottom panel of Fig. 2(a), and is overlaid with three sets of calculated resonance frequencies. The first set consists of the resonance frequencies calculated from the neutral area function, Ω(*i*), and are shown as the thin, static, horizontal lines extending from the beginning to the end of the sentence. These are the reference values for the deflections imposed by the RDP specifications. A second set, shown as thick gray lines, represents a special case for which only the vowel events in the sentence were allowed to influence the area function (i.e., *μ* values were set to zero for all consonant events). These show how the resonances are deflected away from the horizontal lines (neutral resonances) according to the specified RDPs. The third set, shown as black dots, tracks the resonances generated from the time-varying area function with all vowel and consonant events included. The breaks indicate time intervals during which the vocal tract was fully occluded or nearly so; these lines also track the formant frequencies in the wideband spectrogram. Viewing the thick gray lines (vowel events only) along with the black dots (all events) shows the relative and coarticulated nature of the overlapped events. For example, between about 0.7–0.9 s both *f_R_*_2_ and *f_R_*_3_ are sweeping upward in frequency due to the {eh}-{ih} diphthong events, but the RDP for the subsequent {t} also specifies an upward deflection of the same two resonances. The model does indeed assure that both *f_R_*_2_ and *f_R_*_3_ are deflected above those of the diphthong alone, even though they were already deflected well above the resonances of the neutral vocal tract shape Ω(*i*).

An audio file of the synthesized sentence and a slow-motion animation of the time-varying vocal tract shape are available as multimedia files Mm. 1 and Mm. 2, respectively. The vocal tract animation is a projection of the equivalent radii of the time-varying area function onto a 2D profile (Story *et al.*, 2018), and the inset plot shows the calculated resonance frequencies.

**Mm. 1.** Synthesized sentence “*a dog ate a bug.*” This is a file of type “wav” (142 Kb).

**Mm. 2.** Animation of the time-varying vocal tract and resonance frequencies for the sentence “*a dog ate a bug*.” This is a file of type “mov” (1 Mb).

### B. Sentence 2

Figures 2(c) and 2(d) show event functions, time-varying area function, spectrogram, and calculated resonances for the second synthesized sentence, “*a frog ate a fly*.” The total duration is the same as the first sentence, and the temporal characteristics of the events {ao}, {g}, {eh}, {ih}, {t} are either the same or quite similar (some slight adjustments were made to accomodate different consonant events). The two unstressed neutral vowels {ax} are again produced during the absence of any other specified event. What is different from the first sentence is that the first and second events specify a cluster consisting of the fricative {f} and liquid {r}, and the eighth and ninth events specify a similar cluster of {f} and {l} followed by the diphthong {aa}-{ih} (see Table I for timing parameters). The RDP for each {f} deflects the first two resonances downward and, with *μ* = 0.98, will almost fully occlude the vocal tract, but not quite, as is needed for a fricative consonant. In addition, the event functions for both {f}'s include a 0.03 s hold duration (*t_h_*) at the peak value to generate a fricative sound. The liquids were specified primarily by *δ*_3_, which was set to −1 for {r} and +1 for {l}. The other two parameters had the same value for both liquids and were set to *δ*_1_ = –0.3 and *δ*_2_ = 0. The value of *μ* was set to 0.8 for {r} and 0.9 for {l}, both of which generate a large deflection of the third resonance, but a less severe constriction of the vocal tract than the fricative {f}.

The time-varying area function in Fig. 2(d) shows that the primary constriction generated for both {f}'s is essentially located at the lips, the location expected for a speech sound typically produced by contacting the upper incisors with the lower lip (i.e., “labio-dental”). As the first fricative blends into the {r}, two constrictions appear in the area function, one just anterior of hard-palate/soft palate junction (point C), and the other near the superior aspect of the epiglottis (point D). Similarly, the {l} that is produced around 1.2 s also contains two constrictions, but located just posterior to the incisors (point B) and posterior to hard-palate/soft-palate junction (point C), respectively.

The speech signal was generated in the same manner as the first sentence, but with two additional abductory maneuvers of the vocal folds to assure that the fricatives were unvoiced. The spectrogram [Fig. 2(a)] shows frication noise at about 0.14 and 1.16 s for the two {f}'s, respectively, followed by a lowering of the third resonance frequency for the {r} and raising of the same resonance for the {l}. Synthesis of “a frog ate a fly” and corresponding vocal tract animation are available as multimedia files Mm. 3 and Mm. 4, respectively.

**Mm. 3.** Synthesized sentence “*a frog ate a fly.*” This is a file of type “wav” (142 Kb).

**Mm. 4.** Animation of the time-varying vocal tract and resonance frequencies for the sentence “*a frog ate a fly*.” This is a file of type “mov” (1 Mb).

## V. Discussion and conclusion

The model described here was shown to accept, as input, discrete, relative specifications of acoustic speech events and transform them into modulations of the vocal tract to produce sentence-level speech. Although the two sentences synthesized for this study (included as multimedia files) are likely intelligible to many listeners, there are undoubtedly some segments that may sound unusual relative to natural human speech. This is largely due to the heuristic approach taken with regard to using the model. Other than estimating overall sentence duration, neither of the synthesized sentences were, in any way, based on analysis of audio recordings of human speech production. Rather, the events, as shown in Fig. 2, were laid out along a timeline and manually adjusted until a version of each sentence was deemed reasonable by informal listening. Most difficult, and perhaps noticeable from the audio files, was setting the timing of both the vocal tract and laryngeal events for the fricative-liquid clusters in the second sentence in order to generate a plausible voiceless {f} followed by either the {r} or {l}, which, of course, are both voiced.

A next step is to perform perceptual experiments that explore listeners' sensitivity to variations in the RDP values and timing of events. For example, all of the stop consonants in the two synthesized sentences were specified by a set of 1's with either a positive or negative polarity. Perhaps those same consonants could be more naturally generated with magnitudes less than 1.0 depending on the surrounding vowel context. That is, coarticulation may be more naturally produced with flexibility in the magnitudes of the RDPs. Also, considering that each vowel event is a syllable nucleus, it would be of interest to understand how much variability can be imposed on their temporal locations and still retain the same perceptual response. The effects of compressing or expanding the acoustic events in time on the resulting vocal tract modulations could provide additional insights into articulatory variability due to speech rate.

The vocal tract modulations generated by the model produced constrictions and expansions at locations along the vocal tract length axis that are roughly similar to those expected based on general knowledge of articulation, even though an utterance was *planned* entirely by specifying the deflection patterns of the vocal tract resonances. The output of the model, however, both in terms of time-varying area functions and speech waveforms, also needs to be compared to articulatory (articulography, MRI, etc.) and acoustic data collected from human talkers. This will allow for an evaluation of whether the vocal tract modulations are physiologically realistic in a wide variety of phonetic contexts.

Although the model demonstrated here was based on an adult male speech production system, the process of planning an utterance by specifying relative acoustic events along a time line is independent of the talker. This means that the same two sentences generated in this study (or other words, phrases, and sentences) could be produced with qualities of a completely different talker (e.g, sex, age, size, etc.) simply by substituting a different vocal tract and voice source. Of interest would be whether the speech production system of a variety of talkers generates the same or different vocal tract modulations for the same set of acoustic events. The model is also independent of language. The two sentences synthesized by the model were English; however, this is only the case because the acoustic events were arranged according to the phonological rules of English. Sentences in another language could be generated by using a different set of phonological rules.
